# Rationally Designed Synthetic Haptens to Generate Anti-Ciguatoxin Monoclonal Antibodies, and Development of a Practical Sandwich ELISA to Detect Ciguatoxins

**DOI:** 10.3390/toxins11090533

**Published:** 2019-09-13

**Authors:** Takeshi Tsumuraya, Masahiro Hirama

**Affiliations:** Department of Biological Science, Graduate School of Science, Osaka Prefecture University, 1-2, Gakuen-cho, Naka-ku, Sakai, Osaka 599-8570, Japan; masahiro.hirama.c2@tohoku.ac.jp

**Keywords:** ciguatera, ciguatoxin, monoclonal antibody, sandwich ELISA

## Abstract

“Ciguatera” fish poisoning (CFP) is one of the well-known food poisoning caused by the ingestion of fish that have accumulated trace amounts of ciguatoxins (CTXs). CFP affects more than 50,000 individuals annually. The difficulty in preventing CFP comes from the lack of reliable methods for analysis of CTXs in contaminated fish, together with the normal appearance, taste, and smell of CTX-contaminated fish. Thus, a sensitive, accurate, routine, and portable analytical method to detect CTXs is urgently required. Monoclonal antibodies (mAbs) specific against either wing of major CTX congeners (CTX1B, 54-deoxyCTX1B, CTX3C, and 51-hydroxyCTX3C) were generated by immunizing mice with rationally designed synthetic haptens-KLH conjugates instead of the CTXs. Haptenic groups with a surface area greater than 400 Å^2^ are required to produce mAbs that can strongly bind to CTXs. Furthermore, a highly sensitive fluorescence-based sandwich enzyme-linked immunosorbent assay (ELISA) was developed. This protocol can detect and quantify four major CTX congeners (CTX1B, 54-deoxyCTX1B, CTX3C, and 51-hydroxyCTX3C) with a limit of detection (LOD) of less than 1 pg/mL. The LOD determined for this sandwich ELISA is sufficient to detect CTX1B-contaminated fish at the FDA guidance level of 0.01 ppb.

## 1. Introduction

“Ciguatera” fish poisoning (CFP) is one of the well-known food-borne diseases caused by the ingestion of various reef fish that have accumulated trace amounts of ciguatoxins (CTXs) [1,2]. Typical human symptoms of CFP are severe neurological, gastrointestinal, and cardiovascular symptoms, which may last week, months, or even years. It is estimated that over 50,000 people throughout the world suffer from CFP each year, making it the most common form of seafood poisoning caused by natural toxins [3,4,5]. The spread of CFP gives rise to a considerable burden on public health systems, and damage to fishery resources and the economies in tropical and subtropical regions [6]. In addition to climate change, globalization of trade might contribute to the expansion of CFP, even in temperate zones. It is difficult to prevent CFP due to the lack of reliable analytical methods to detect causative CTXs, along with the normal appearance, taste, and smell of CTX-contaminated fish. 

**Figure 1:**
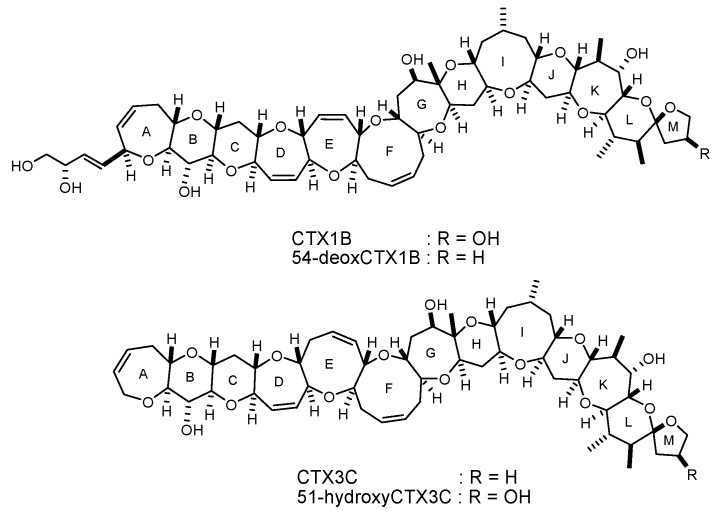
Chemical structures of the four major ciguatoxin (CTX) congeners: CTX1B, 54-deoxyCTX1B, CTX3C, and 51-hydroxyCTX3C.

Yasumoto and his coworkers characterized CTXs as 3 nm-long ladder-shaped polycyclic ethers. CTXs were originally produced by *Gambierdiscus* species of marine dinoflagellates and accumulate in various types of reef fish through bioaccumulation [1,2,7]. CTX1B and its congeners (CTX1B, 54-deoxyCTX1B, CTX3C, and 51-hydroxyCTX3C) (Figure 1) were initially isolated in the Pacific region [8,9,10,11,12], then detected in the Atlantic [13,14]. These CTXs are extremely toxic to mammals and the lethal potencies of CTXs by intraperitoneal injection into mice (median lethal dose (LD_50_) 0.25–4 μg/kg) are much greater than those of the structurally related red-tide toxins, brevetoxins (LD_50_ > 100 μg/kg) and the pufferfish toxin, tetrodotoxin (LD_50_~10 μg/kg) [8,9,10,15,16,17]. The structural differences between the CTX congeners arise from the substituents on the A and M terminal rings as well as the size of the E-ring. The CTX1B series (CTX1B and 54-deoxyCTX1B) possesses a dihydroxybutenyl group on the A-ring and a seven-membered E-ring, while members the CTX3C series (CTX3C and 51-hydroxyCTX3C) lack the dihydroxybutenyl group and have an eight-membered E-ring.

**Figure 2:**
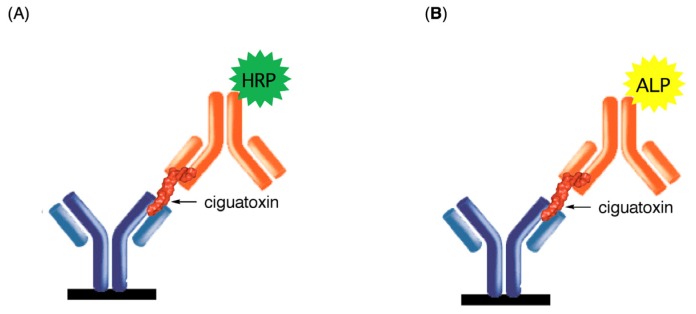
Schematic representation of the detection of CTXs by sandwich ELISA. Monoclonal antibodies (mAbs) (blue) against the left end of CTXs (red) is adsorbed on the wells of a 96-well microtiter plate and mAb (orange) against the right end is labeled with (**A**) horseradish peroxidase (HRP, green) or (**B**) alkaline phosphatase (ALP, yellow).

Considerable attention has recently been directed to the development of analytical methods for detecting CTXs. To replace the traditional mouse bioassay (MBA), several analytical methods have been developed in recent years to detect CTXs in a contaminated fish. Examples include a neuroblastoma cell-based assay (CBA-N2a) [18], radiolabeled and fluorescent receptor binding assays (RBA) [16,19,20], HPLC [21], MS [12,22,23], and LC-MS/MS assays [13,14,24,25,26,27,28,29]. However, there is no quick and reliable method to detect CTXs in the fishery or even at inspection stations for seafood. MAb-based immunoassays such as ELISA was expected to provide suitable methods for the sensitive, accurate, routine, and portable detection of CTXs. For the generation of anti-CTX antibodies, Hokama et al. claimed that an anti-CTX1B mAb was prepared by immunization with natural CTX1B [30], but the mAb cross-reacted with okadaic acid (OA) [31,32], and the results of immunochemical kit “Cigua-Check^®^” using this mAb have been controversial [33,34,35]. The minimal amount of CTX isolated from contaminated fish has prevented further development of anti-CTX antibodies. Thus, rationally designed synthetic haptens instead of natural toxins were planned to produce anti-CTX mAbs. Successful syntheses of CTX congeners based on a convergent synthetic strategy unifying right wing (ABCDE) and left wing (HIJKLM) fragments [36,37,38,39,40] allowed to synthesize nontoxic haptens to generate mAbs that recognize the specific structure of CTXs. Here, the current status of the preparation of anti-CTX mAbs and development of highly sensitive sandwich ELISA to detect CTX congeners is summarized (Figure 2) [41,42,43,44,45,46,47,48,49].

## 2. Generation of Anti-CTX MAbs

### 2.1. Design of Haptens and Preparation of Protein-Conjugates

**Figure 3:**
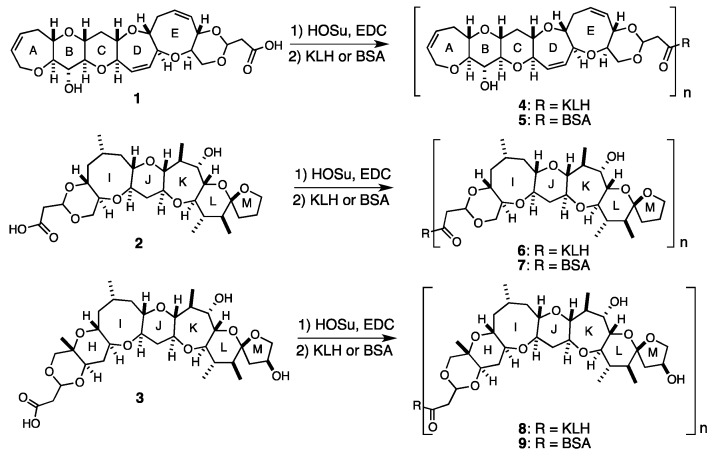
Structures of synthetic haptens **1**–**3** and synthesis of protein-conjugates **4**–**9**.

Since the maximum buried surface areas of small haptens in antibody-hapten complexes are estimated to be approximately 400 Å^2^ [50,51,52,53,54,55,56], it was predicted that hapten **1**, consisting of a pentacyclic skeleton (ABCDE ring, calculated water accessible surface area was 398 Å^2^) and a cyclic acetal connected to a linker, would be sufficiently large to occupy the antibody-combining site, whereas leaving the acetal and linker moiety free from interactions with the antibody-combining site [44]. In addition, hapten **1** was designed to induce mAbs that would bind to the left wing of CTX3C or 51-hydroxyCTX3C in an orientation suitable for sandwich ELISA. Similarly, hapten **2** consisting of a pentacyclic skeleton (IJKLM ring, calculated water accessible surface area was 477 Å^2^) was designed to induce mAbs against the right wing of CTX3C and 54-deoxyCTX1B [44]. Hapten **3** bearing a hydroxy group on the M ring was designed to generate mAbs for the right wing of 51-hydroxyCTX3C and CTX1B [45]. Since small-molecular-weight haptens such as **1**, **2**, and **3** can be made immunogenic only by coupling them to carrier proteins, these haptens were linked with keyhole limpet hemocyanin (KLH) and also with bovine serum albumin (BSA) via a conventional active ester method using *N*-hydroxysuccimide (HOSu) and 1-ethyl-3-(3-dimethylaminopropyl) carbodiimide (EDC) to provide the KLH- and BSA-conjugates **4**–**9** (Figure 3). The KLH-conjugates **4**, **6**, and **8** were used for immunization and the BSA-conjugates **5**, **7**, and **9** were used for the binding analysis of hybridoma supernatants by ELISA.

In contrast, the conjugation of synthetic haptens smaller than **1**, **2**, and **3** with KLH did not elicit competent mAbs [41,42,43] showing that pentacyclic haptens of more than 400 Å^2^ surface areas are required to elicit mAbs binding strongly to CTXs.

**Figure 4:**
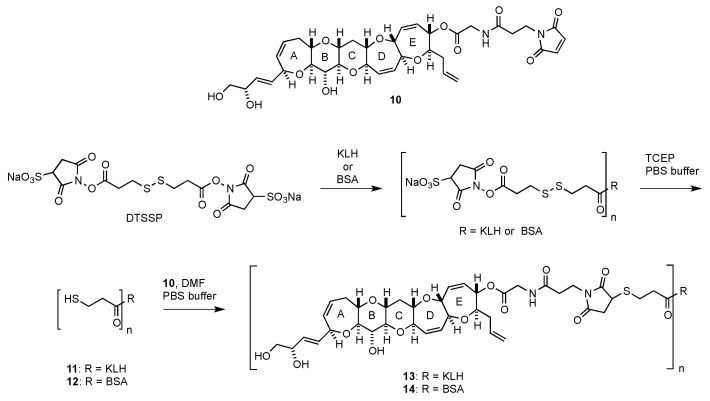
Structure of hapten **10** and synthesis of protein-conjugates **13** and **14** for the left wing of CTX1B and 54-deoxyCTX1B.

However, it was difficult to raise mAbs against the left wing of the CTX1B series possessing a dihydroxybutenyl group on the A-ring (CTX1B and 54-deoxyCTX1B). After several unsuccessful immunization studies using synthetic hapten-KLH conjugates connected through acetal linkers [41], the conjugation strategy was changed and the pentacyclic hapten **10** was synthesized to prepare mAbs against the left wing of the CTX1B series [47]. The KLH- and BSA-conjugates **13** and **14** were reliably synthesized from maleimide **10**, as outlined in Figure 4. KLH and BSA were converted to thiol-enriched KLH **11** and BSA **12**, respectively, using 3,3’-dithiobis (sulfosuccinimidyl propionate) (DTSSP) followed by reduction with tris (2-carboxyethyl) phosphine (TCEP). The coupling of **10** with **11** and **12** yielded KLH- and BSA-conjugates **13** and **14**, respectively.

### 2.2. Production and Affinity Measurement of Anti-CTX mAbs

MAbs specific to the left wing of the CTX3C series (CTX3C and 51-hydroxyCTX3C) were produced by immunization of mice with KLH-conjugate **4** according to the standard protocol. After three injections of KLH-conjugate **4**, the titer of serum immunoglobulin G (IgG) was determined by ELISA using BSA-conjugate **5**. The mouse showing the highest titer was boosted; after 3 days, the spleen was taken from the mouse, and the splenocytes were fused with X63-Ag8.653 myeloma cells using an electro cell fusion apparatus as reported previously [53,54]. All hybridomas producing IgGs that bound to BSA-conjugate **5** were subcloned twice or three times to provide six hybridomas producing BSA-conjugate **5**-binding mAbs. Competitive ELISA analyses of hybridoma supernatants showed that one mAb, designated 10C9, was most strongly inhibited by ABCDE fragment **15** and thus 10C9 was chosen for further analysis [44].

Similarly, immunization with KLH-conjugate **6**, followed by competitive ELISA with IJKLM-fragment **16**, provided mAb 3D11 that specifically bound the right wing of CTX3C and 54-deoxy CTX1B [44].

MAbs specific for the right wing of CTX1B and 51-hydroxyCTX3C were prepared by immunization with KLH-conjugate **8** to elicit mAbs (7C9, 8B9, and 8H4) whose dissociation constants (*K*_d_s) for HIJKLM fragment **17** determined by competitive ELISA were 407, 108, and 48 nM, respectively. Thus, mAb 8H4 showing the highest affinity was selected for further studies [45].

KLH-conjugate **13** was immunized to prepare mAbs specific for the left wing of the CTX1B series (CTX1B and 54-deoxyCTX1B). This immunization provided 18 cell lines producing mAbs that bound to BSA-conjugate **14**. Competitive ELISA analyses of hybridoma supernatants showed that the binding of mAb 3G8 was strongly inhibited by 0.1 μM ABCDE fragment **18** and thus 3G8 was selected for further analysis [47].

**Figure 5:**
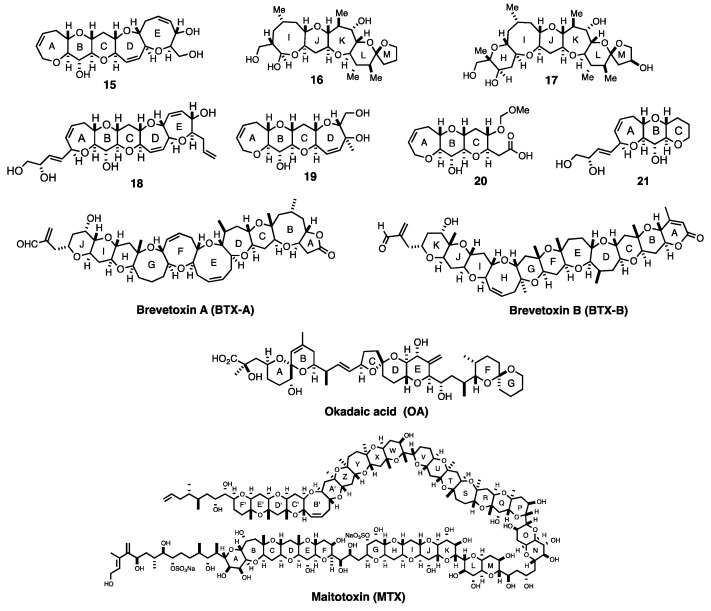
Structures of synthetic fragments **15**–**21** and structurally related marine toxins.

To evaluate the molecular recognition and interaction of four anti-CTX mAbs 10C9, 3D11, 8H4, and 3G8, the *K*_d_s of these mAbs against four CTXs, as well as against small synthetic fragments and structurally related marine toxins, were determined by competitive ELISA (Table 1) [57,58]. As expected, mAb 10C9 bound strongly to ABCDE ring fragment **15** with a dissociation constant (*K*_d_) of 0.8 nM (Figure 5). More importantly, 10C9 bound also to CTX3C itself, with a comparable *K*_d_ of 2.8 nM. Mab 10C9 showed no cross-reactivity with either the other-wing fragment (IJKLM ring **16**) or with the structurally related marine toxins such as brevetoxin A (BTX-A) [59,60], brevetoxin B (BTX-B) [61,62], okadaic acid (OA) [63], and maitotoxin (MTX) [64]. Furthermore, the *K*_d_ values of mAb 10C9 for tetracyclic fragment (ABCD ring **19**) and tricyclic fragment (ABC ring **20**) were determined to be 1.8 and 74 μM, respectively, while that for pentacyclic fragment (ABCDE ring **15**) was 0.8 nM (Table 1) [44]. Thus, as the synthetic fragments become smaller, the *K*_d_ value of 10C9 increased 2250-fold (5 to 4 rings) or 41-fold (4 to 3 rings). This result suggests that mAb 10C9 recognizes the entire pentacyclic skeleton (ABCDE ring) of CTX3C, and that the surface area required for molecular recognition of 10C9 is consistent with the prediction of 400 Å^2^. Probable recognition of mAb 10C9 and the pentacyclic ring moiety (ABCDE ring) of CTX3C was confirmed by crystal structure analysis of 10C9 complexed with ABCDE ring **15 [65]**. 

Similarly, mAb 3D11 also bound strongly to both IJKLM ring **16** (*K*_d_ = 8.6 nM) and CTX3C (*K*_d_ = 122 nM), and did not show any cross-reactivity with synthetic fragment (ABCDE ring **15**) or other marine toxins (Table 1) [44].

MAb 8H4 strongly bound to both CTX1B and 51-hydroxyCTX3C with *K*_d_s of 20.4 and 13.6 nM, respectively. The *K*_d_ value of 8H4 for CTX3C, which lacks an M-ring hydroxyl group, was 3.2 μM. These results indicate that 8H4 distinguishes between CTXs with and without an M-ring hydroxyl group. This mAb also did not cross-react with the structurally related cyclic ethers BTX-A, BTX-B, OA, and MTX [45].

MAb 3G8 was found to bind very strongly to ABCDE ring fragment **18**, with a *K*_d_ of 1.5 nM, and also strongly to CTX1B with a *K*_d_ of 15 nM. 3G8 did not cross-react with CTX3C, which lacks a dihydroxybutenyl substituent on the A ring, indicating that mAb 3G8 recognizes the A-ring dihydroxybutenyl substituent. In addition, the *K*_d_ of 3G8 for ABC-ring fragment **21** was 410 nM, 270 times larger *K*_d_ than that for ABCDE ring fragment **18**, indicating that mAb 3G8 recognizes the whole pentacyclic ring skeleton (ABCDE ring) of CTX1B [47]. Since CTX1B and 54-deoxyCTX1B share the left end, mAb 3G8 should also bind to 54-deoxyCTX1B. This was confirmed by the successful sandwich ELISA detection of 54-deoxyCTX1B using mAbs 3G8 and 8H4 [49]. MAb 3G8 did not cross-react with the structurally related marine toxins.

## 3. Sandwich ELISA Detection of CTXs

**Figure 6:**
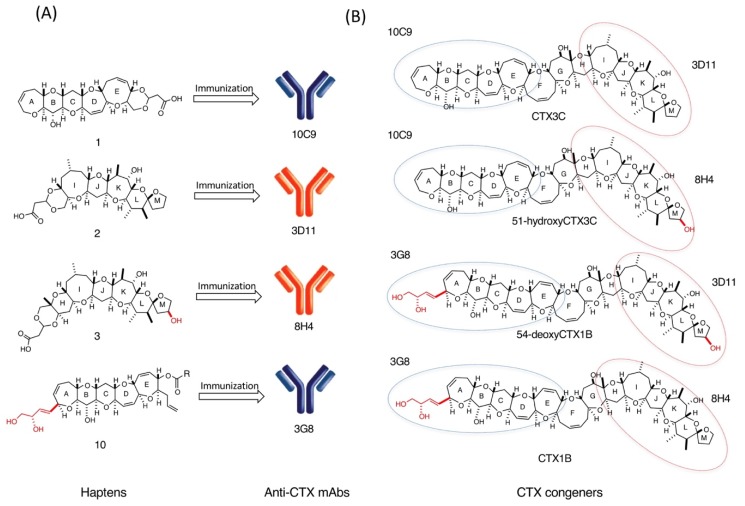
Summary of anti-CTX mAbs produced by immunization with synthetic haptens (**A**) and their combination for detection of four CTX major congeners by sandwich ELISA (**B**). The circles in figure (**B**) indicate the wings of the CTX congeners to which each mAb binds.

**Figure 7:**
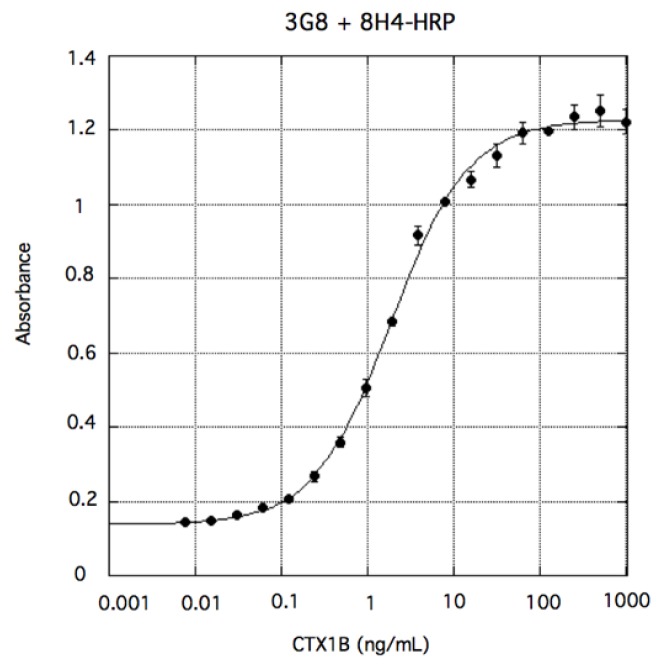
Detection of CTX1B by sandwich ELISA using 3G8 and HRP-labeled 8H4. The values represent mean absorbance (λ = 490 nm) of three experiments. The error bars represent standard deviations.

Four anti-CTX mAbs that bind strongly and specifically to CTXs are now available, as summarized in Figure 6. 10C9 binds to the left end of the CTX3C series (CTX3C and 51-hydroxyCTX3C) while 3D11 binds to the right end of CTX3C and 54-deoxyCTX1B. Similarly, 3G8 binds to the left end of the CTX1B series (CTX1B and 54-deoxyCTX1B) whereas 8H4 binds to the right end of CTX1B and 51-hydroxyCTX3C. Using two of these anti-CTX mAbs, a sandwich ELISA was developed for the detection of the major CTX congeners (Figure 2 and Figure 6). First attempted was detection of CTXs by sandwich ELISA using a combination of two mAbs: a mAb for the left end (10C9 or 3G8) was adsorbed on a microtiter plate as a capture antibody (Ab) and a horseradish peroxidase (HRP)-labeled mAb for the right end (3D11-HRP or 8H4-HRP) was used as a detection Ab. For example, to detect CTX1B, 3G8 was used to capture CTX1B and 8H4 as a detection Ab. 96-well microtiter plate wells were directly coated with 3G8 whereas 8H4 was labeled with HRP. In the sandwich ELISA *o*-pheylenediamine (OPD) was used as a colorimetric substrate to detect CTX1B specifically as shown in Figure 7 [47]. The limit of detection (LOD) was 0.28 ng/mL. Similar sandwich ELISA protocols also detected other CTXs, CTX3C, 51-hydroxyCTX3C, and 54-deoxyCTX1B: 10C9 and HRP-linked 3D11 were used to detect CTX3C [44], 10C9 and HRP-linked 8H4 were used to detect 51-hydroxyCTX3C [45], and 3G8 and HRP-linked 3D11 were used to detect 54-deoxyCTX1B. The cross-reactivities of the sandwich ELISA were also investigated against structurally related marine natural toxins, such as BTX-A, BTX-B, OA, and MTX. The sandwich ELISA did not show any cross-reactivity with these marine toxins [44,45,47].

## 4. Highly Sensitive Detection of CTXs

### 4.1. Fluorescent Sandwich ELISA Detection of CTXs

**Figure 8:**
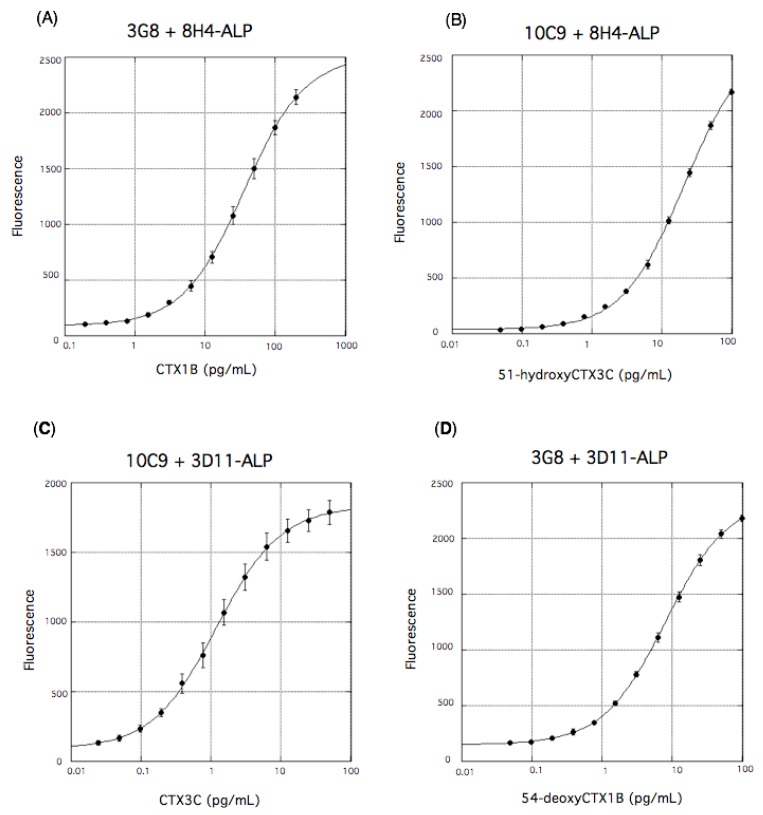
Fluorescent sandwich ELISA detection of CTX1B using 3G8 and ALP-labeled 8H4 (**A**), 51-hydroxyCTX3C using 10C9 and ALP-labeled 8H4 (**B**), CTX3C using 10C9 and ALP-labeled 3D11 (**C**), and 54-deoxyCTX1B using 3G8 and ALP-labeled 3D11 (**D**). Immunoreaction enhancer solution (Can Get Signal^®^) was added to detect CTX3C (**C**) and 54-deoxyCTX1B (**D**). The values represent mean fluorescence intensities (excitation, λ = 435 nm; emission, λ = 555 nm) of three experiments. The error bars represent standard deviations.

Although a regulatory limit for CTXs in fish has not been established by the United Nations the World Health Organization (WHO) and Food and Agriculture Organization (FAO), the United States Food and Drug Administration (FDA) issued a guidance level of 0.01 ppb CTX1B equivalent toxicity in fish [5,66]. Therefore, the sensitivity of the sandwich ELISA should be adequate to reliably detect CTX1B at the FDA guidance level (0.01 ppb) in contaminated fish. The sensitivity of 0.28 ng/mL observed in the HRP-based sandwich ELISA detection of CTX1B is thus inadequate and so a protocol to improve the sensitivity in the sandwich ELISA was investigated. The combination of alkaline phosphatase (ALP) as a conjugated enzyme and 2’-(2-benzothiazoyl)-6’-hydroxybenzothiazole phosphate (BBTP) as a fluorogenic substrate is optimal (Figure 8). The BBT anion is produced by the reaction of ALP with BBTP and has a large Stokes’ shift, which leads to lower background fluorescence as well as higher detection sensitivity. This new fluorescent sandwich ELISA can detect CTXs with extremely high sensitivity (vide infra) [49].

Capture Ab 3G8 was adsorbed on the wells of 96-well microtiter plates and detection Ab 8H4 was labeled with ALP. A general sandwich ELISA protocol using a fluorescent substrate BBTP allowed detection of CTX1B as shown in Figure 8A. The detection sensitivity of this fluorescent sandwich ELISA was improved considerably and the LOD was determined to be 0.16 pg/mL. Similar fluorescent sandwich ELISA using mAb 10C9 and ALP-labeled 8H4 allowed a sensitive detection of 51-hydroxyCTX3C with an LOD of 0.1 pg/mL (Figure 8B). However, fluorescent sandwich ELISA using ALP-labeled 3D11 for the right wing gave unsatisfactory results: sandwich ELISA using mAb 10C9 for the left end and ALP-labeled 3D11 for the right end gave an unsatisfactory LOD for CTX3C (ca. 2 pg/mL); in addition, the background was high, probably due to the relatively weak binding of 3D11 to CTX3C (*K*_d_ = 122 nM) [44]. Thus, to improve the detection sensitivity, immunoreaction enhancer solution (Can Get Signal^®^) was added to the fluorescent sandwich ELISA system. This solution significantly improved the LOD (0.090 pg/mL, Figure 8C) and lowered the background fluorescence in the detection of CTX3C. The addition of this additive to the fluorescent sandwich ELISA using 3G8 and ALP-labeled 3D11 also led to a significant improvement in the detection of 54-deoxyCTX1B with an LOD of 0.11 pg/mL (Figure 8D). In contrast, this enhancer solution did not improve the LOD or the background in fluorescent sandwich ELISAs using 3G8 and ALP-labeled 8H4 to detect CTX1B and 10C9 and ALP-labeled 8H4 to detect 51-hydroxyCTX3C [49].

### 4.2. Cross-Reactivity of the Fluorescent Sandwich ELISA

The cross-reactivities of the present fluorescent sandwich ELISA protocols were first investigated against other related marine toxins, such as BTX-A, BTX-B, OA, and MTX. The sandwich ELISA showed high specificity to CTXs and did not show any cross-reactivity with the structurally related marine toxins [49].

**Figure 9:**
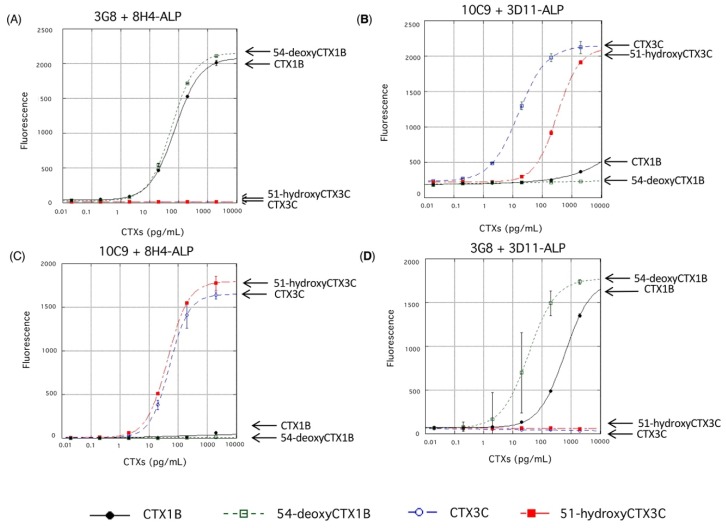
Cross-reactivity of fluorescent sandwich ELISA against the four major CTX congeners (CTX1B, 54-deoxyCTX1B, CTX3C, and 51-hydroxyCTX3C). (**A**) Cross-reactivity of the ELISA originally designed for the detection of CTX1B using 3G8 and ALP-labeled 8H4. (**B**) Cross-reactivity of the ELISA originally designed for the detection of CTX3C using 10C9 and ALP-labeled 3D11. (**C**) Cross-reactivity of the ELISA originally designed for the detection of 51-hydroxyCTX3C using 10C9 and ALP-labeled 8H4. (**D**) Cross-reactivity of the ELISA originally designed for the detection of 54-deoxyCTX1B using 3G8 and ALP-labeled 3D11. The values represent mean fluorescence intensities (excitation, λ = 435 nm; emission, λ = 555 nm) of three experiments. The error bars represent standard deviations.

Next, the cross-reactivity of the fluorescent sandwich ELISA against the other congeners of CTX were examined (Figure 9). Each ELISA protocol strictly differentiate between the CTX1B series and the CTX3C series, namely between CTX congeners with and without the dihydroxybutenyl group on the A ring. Thus, 3G8 specifically recognizes the presence of the dihydroxybutenyl group on the A ring of CTX1B and 54-deoxyCTX1B (Figure 9A,D), whereas 10C9 strictly recognizes the A ring that lacks the substituent in CTX3C and 51-hydroxyCTX3C (Figure 9B,C). 

More interesting is that ALP-labeled 8H4 appears to have tolerance for the absence of the hydroxy group on the M ring in sandwich ELISA (CTX1B vs. 54-deoxyCTX1B in Figure 9A; CTX3C vs. 51-hydroxyCTX3C in Figure 9C), while free 8H4 discriminated more strictly 51-hydroxyCTX3C (*K*_d_ = 13.8 nM) from CTX3C (*K*_d_ = 3.2 μM) in an aqueous media [45]. Meanwhile, ALP-labeled 3D11 still showed some discrimination (Figure 9B,D). Based on the above results ALP-labeled 8H4, rather than ALP-labeled 3D11, should be used for a fluorescent sandwich ELISA designed to bind the right end of CTXs regardless of the presence or absence of the hydroxy group on the M ring as shown in the following section [49].

### 4.3. Single ELISA Analysis of Four CTX Congeners 

**Figure 10:**
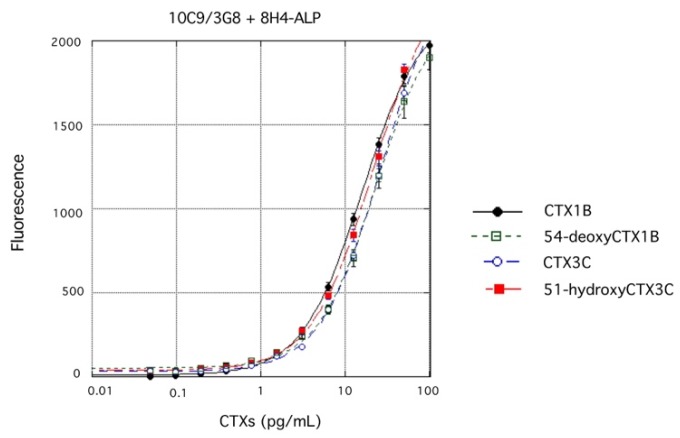
Sandwich ELISA detection of any of four CTX congeners (CTX1B, 54-deoxyCTX1B, CTX3C, and 51-hydroxyCTX3C) using one microtiter plate whose wells were coated with a 1:1 mixture of capture Abs 10C9 and 3G8, ALP-labeled 8H4, and BBTP. The values represent mean fluorescence intensities (excitation, λ = 435 nm; emission, λ = 555 nm) of three experiments. The error bars represent standard deviations.

For practical purposes, it is crucial to detect CTXs irrespective of the congener since several CTX congeners are typically present in a contaminated fish. A more convenient sandwich ELISA to detect any of the four CTXs (CTX1B, 54-deoxyCTX1B, CTX3C, and 51-hydroxyCTX3C) was developed using one microtiter plate in a single operation: a 1:1 mixture of 10C9 and 3G8 was adsorbed on the surface of wells of a microtiter plate, while ALP-labeled 8H4 and BBTP were used for detection. All four CTX congeners were detected effectively (Figure 10). It is noteworthy that these four CTXs gave similar fluorescence intensities as the concentrations of CTX changed, indicating that this sandwich ELISA protocol can detect and quantify any of the four CTX congeners in a single operation [49]. 

### 4.4. Detection of CTX1B from Fish Flesh and Evaluation of the Matrix Effects at Each Extraction/Purification Step

**Figure 11:**
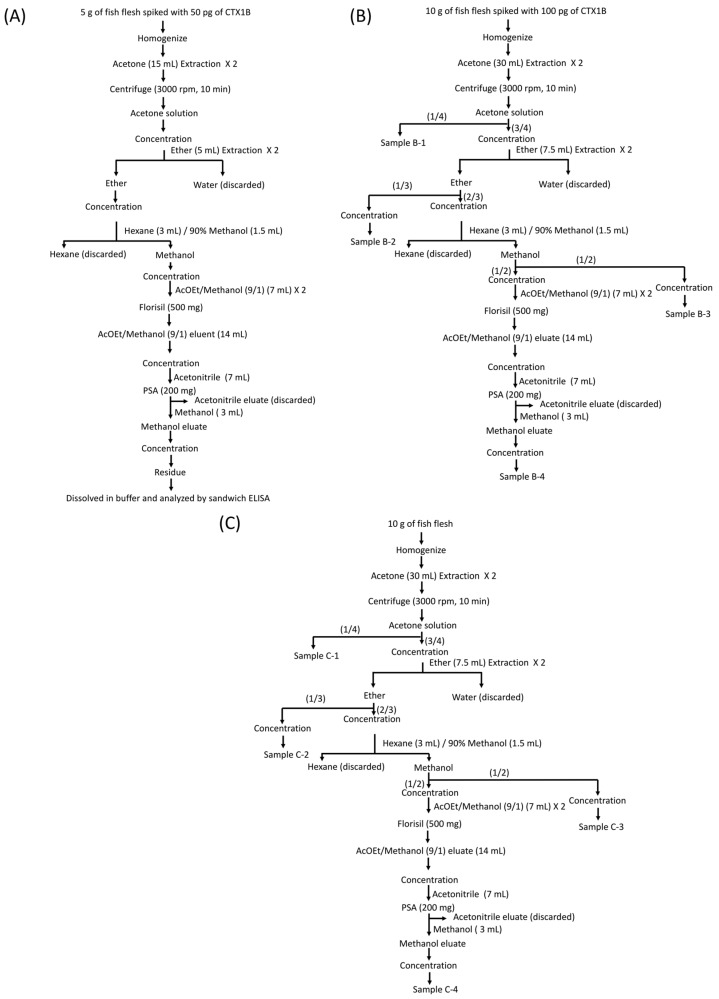
(**A**) Extraction and purification scheme for CTX1B. Fish flesh was spiked with Food and Drug Administration (FDA) guidance level (0.01 ppb) of pure CTX1B and after extraction/purification, the sample and the tenfold dilution were analyzed by fluorescent sandwich ELISA. (**B**) Extraction and purification scheme for the evaluation of matrix effects at each step of the extraction/purification procedure. 0.01 ppb of pure CTX1B was spiked into fish flesh and after extraction/purification each extract (Samples B-1~B-4) was analyzed by fluorescent sandwich ELISA. (**C**) Extraction and purification scheme for direct evaluation of the matrix effects of each extract. 0.01 ppb of pure CTX1B was added to each extract obtained by the extraction/purification procedure (Samples C-1~C-4) and each sample was analyzed by fluorescent sandwich ELISA.

To confirm that the fluorescent sandwich ELISA is applicable to the detection of CTX1B at the FDA guidance level, fish (*Variola louti*) determined to be free of CTXs by LC-MS/MS analysis [25], was spiked with pure CTX1B (0.01 ppb), the flesh was extracted with acetone, and the extract was purified as shown in Figure 11A. The extraction/purification method was originally developed for the analysis of CTXs by LC-MS/MS [25]. The CTX1B concentrations determined by the fluorescent sandwich ELISA in the final extract and its 10-fold dilution were 17 pg/mL and 20 pg/mL, respectively, after calculation using the dilution factor. These results showed that 34% and 40% of the initially spiked CTX1B was detected. Thus, CTX1B spiked into the flesh of fish at the FDA guidance level of 0.01 ppb was securely detected by the fluorescent sandwich ELISA protocol after extraction/purification using a conventional procedure [49].

To simplify the extraction/purification procedure, the recovery of CTX1B was then evaluated at every extraction/purification step, because contaminants like lipids and proteins in fish flesh may have an effect on the fluorescent sandwich ELISA assay through their matrix effects. Ten grams of flesh of *Variola louti* was spiked with CTX1B (100 pg), which is equal to the amount of the FDA guidance level (0.01 ppb). At each extraction/purification step, a portion of the sample was collected for analysis by sandwich ELISA, as shown in Figure 11B. To each sample of B-1~B-4 was added 1 mL of analysis buffer and then each sample was analyzed by sandwich ELISA. The CTX1B concentrations were determined to be 2.3 pg/mL for sample B-1, 2.5 pg/mL for sample B-2, 2.7 pg/mL for sample B-3, and 8.0 pg/mL for sample B-4. The results indicated that 9%, 10%, 11%, and 32% were recovered from the initially spiked CTX1B for samples B-1, B-2, B-3, and B-4, respectively. In the earlier steps of the extraction/purification procedure, the concentrations of CTX1B determined by sandwich ELISA were significantly decreased to about 10% of the initially spiked CTX1B, probably due to the matrix effects [49]. 

Furthermore, to evaluate the matrix effects of contaminants on the fluorescent sandwich ELISA more directly, extracts were prepared from 10 g of fish flesh (*Variola louti*) following the above-mentioned procedure without adding CTX1B (Figure 11C), then a solution of pure CTX1B (25 pg) in analysis buffer (1 mL) was added to each extract (samples C-1~C-4 in Figure 11C) before conducting the sandwich ELISA analysis. The concentrations of CTX1B determined by the fluorescent sandwich ELISA were 3.2 pg/mL (13% of added CTX1B) for sample C-1, 5.3 pg/mL (21%) for sample C-2, 7.7 pg/mL (31%) for sample C-3 and 25 pg/mL (100%) for sample C-4. These results clearly showed that the significant matrix effects of contaminants were observed in samples C-1, C-2, and C-3 (in decreasing order). However, due to the high sensitivity of the fluorescent sandwich ELISA, simple extraction by homogenization in acetone and centrifugation is very useful as a pre-treatment procedure to detect CTX1B-contaminated fish at 0.01 ppb (FDA guidance level) [49]. 

Table 2 summarizes the LOD, specificity, pre-treatment of the sample, and sample-to-answer time of the fluorescent sandwich ELISA and other established methods developed for the detection of CTXs. The fluorescent sandwich ELISA is superior in terms of LOD and specificity compared with the other established methods. Although LC-MS/MS has the shortest sample-to-answer time, it should be noted that this is the time needed to analyze one sample, while the sandwich ELISA, receptor binding and cell-based assays can analyze multiple samples in a single operation.

## 5. Conclusions

Recently, great progress has been made in generation of anti-CTX mAbs. Anti-CTX mAbs (10C9, 3D11, 8H4, and 3G8) that specifically bind either wing of CTXs were prepared by immunization with KLH conjugates of designed synthetic haptens instead of scarce natural toxins. The synthetic haptens and the synthetic strategy for preparation of the hapten-KLH conjugates are critical for the production of mAbs that bind strongly and specifically to CTXs. The surface area >400 Å^2^ of the haptenic groups are required to induce mAbs that strongly bind CTXs [44]. It should be emphasized that organic synthesis played a critical role in the development of these specific mAbs against large natural marine toxins containing ladder-like polycyclic ethers. In addition, a highly sensitive and accurate fluorescent sandwich ELISA for major CTX congeners has been developed. The ELISA show no cross-reactivity with other structurally related marine natural toxins. The fluorescent sandwich ELISA can detect and quantify four major congeners of CTX (CTX1B, 54-deoxyCTX1B, CTX3C, and 51-hydroxyCTX3C) with an LOD of <1 pg/mL. The sensitivity observed in this ELISA is sufficient to detect CTX1B-contaminated fish at the FDA guidance level following a conventional extraction/purification procedure. Moreover, the sandwich ELISA using one microtiter plate whose wells were coated with a mixture of two mAbs 10C9 and 3G8 together with ALP-labeled 8H4, can detect any four major congeners of CTXs in a single operation. It is also demonstrated that simple homogenization in acetone followed by centrifugation without any tedious extraction and purification procedures is satisfactory for the detection of CTX1B in fish at the FDA guidance level by the fluorescent sandwich ELISA [49]. The sandwich ELISA would be helpful for epidemiological and physiological studies as well as for the prevention of CFP. A fluorescent sandwich ELISA kit “CTX-ELISA^TM^ 1B” consisting of a 96-well microtiter plate coated with capture mAb 3G8 and ALP-linked 8H4 for detection of the CTX1B series (CTX1B and 54-deoxyCTX1B) can be purchased commercially from Fujifilm Wako Corporation, Japan (382-14341) and another kit for detecting the CTX3C series (CTX3C and 51-hydroxyCTX3C) will be soon available from Fujifilm Wako Corporation, Japan. Portable, easy-to-use ELISA kits for detecting CTX congeners will also be developed. 

## Figures and Tables

**Table 1:** Dissociation constants (*K*_d_s) of anti-CTX mAbs against synthetic fragments and marine toxins ^1^.

mAb	*K*_d_ (nM)										
	15	16	17	18	CTX1B	CTX3C	51-hydroxy-CTX3C	BTX-A	BTX-B	OA	MTX
10C9	0.8	NI	ND	ND	ND	2.8	ND	NI	NI	NI	NI
3D11	NI	8.6	ND	ND	ND	122	ND	43000	NI	NI	NI
8H4	ND	ND	48	ND	20.4	3200	13.6	NI	NI	NI	NI
3G8	ND	ND	ND	1.5	15	NI	ND	NI	NI	NI	ND

^1^ The *K*_d_’s were determined by competitive ELISA. ND: not determined. NI: no inhibition was observed at the maximum concentration of each inhibitor (for 10C9, 250 μM **16**, 100 μM BTX-A, 100 μM BTX-B, 100 μM OA, 25 μM MTX; for 3D11, 250 μM **15**, 100 μM BTX-A, 100 μM BTX-B, 100 μM OA, 25 μM MTX; for 8H4, 100 μM BTX-A, 100 μM BTX-B, 100 μM OA, 25 μM MTX; for 3G8, 1.9 μM CTX3C, 10 μM BTX-A, 100 μM BTX-B, 100 μM OA).

**Table 2:** Comparison of limit of detection (LOD), specificity, sample preparation methods, and sample-to-answer time of the analytical methods developed for the detection of CTXs.

Methods	Fluorescent Sandwich Elisa	LC-MS/MS	Receptor Binding Assay (RBA)	Cell-Based Assay (CBA-N2a)
LOD	0.09–0.16 pg/mL (18–32 pg/kg) ^1^	20 pg/mL (4 ng/kg) ^1^	0.075 ppb (75 ng/kg)	0.002 ppb (2 ng/kg)
Specificity	high	high	none	none
Sample preparation methods	Extraction + solid phase extraction cartridges (Florisil + PSA)	Extraction + solid phase extraction cartridges (Florisil + PSA)	Extraction + solid phase extraction cartridge (C18)	Extraction + solid phase extraction cartridge (C18)
Sample-to-answer time	2.5 h	30 min	<3 h	2.5 days
References	[49]	[25,26]	[16,19,20]	[18,20]

^1^ It was assumed that the sample extracted and purified from fish flesh (5 g) was dissolved in buffer or solvent (1 mL) and then analyzed.
